# Comparison of core genome multi-locus sequencing typing pipelines for hospital outbreak detection of common bacterial pathogens

**DOI:** 10.1128/jcm.00646-25

**Published:** 2025-08-27

**Authors:** Heather L. Glasgow, Ying Zheng, Jessica N. Brazelton, Li Tang, Randall T. Hayden

**Affiliations:** 1 Pathology Department, St. Jude Children's Research Hospital 5417, , Memphis, Tennessee, USA; 2 Biostatistics Department, St. Jude Children's Research Hospital 5417, , Memphis, Tennessee, USA; National Institute of Allergy and Infectious Diseases Division of Intramural Research, Bethesda, Maryland, USA

**Keywords:** outbreak detection, outbreak surveillance, whole genome sequencing, core genome multi-locus sequence typing, bacterial relatedness, strain typing

## Abstract

Microbial whole genome sequencing (WGS)-based methods have replaced conventional methods for genomic relatedness analysis in the investigation of or surveillance for infectious outbreaks. Analysis of WGS by core genome multi-locus sequence typing (cgMLST) has been proposed for standardized strain comparisons at high resolution and for longitudinal outbreak surveillance. We compared three commercial cgMLST software pipelines, Ridom SeqSphere+, 1928 Diagnostics’ platform, and Ares Genetics ARESdb, for the identification of related (clustered) strains among 255 isolates of common bacterial pathogens, including *Acinetobacter baumannii, Escherichia coli, Enterococcus faecalis, Enterococcus faecium, Klebsiella pneumoniae, Pseudomonas aeruginosa, Staphylococcus aureus,* and *Serratia marcescens*. Isolates were previously identified as clustered with at least one other isolate collected from the same patient or different patients. Concordance with SeqSphere+ for differentiating clustered from non-clustered isolate pairs using suggested thresholds was 100% for the 1928 platform and 99.5% for ARESdb overall and 91.8%, 96.1%, and 100% among same-patient clustered, different-patient clustered, and different-patient non-clustered isolate pairs, respectively, in ARESdb. ARESdb showed significantly greater allelic distances than SeqSphere+ and 1928 among same-patient clustered isolate pairs (mean [standard deviation, SD], 7.6 [7.17], 1.18 [1.56], and 1 [1.59]) and different-patient clustered isolate pairs (mean [SD], 8.34 [4.31], 3.61 [2.26], and 3.91 [2.67]) (*P* < 0.0001), but not among non-clustered isolate pairs. For all species analyzed with sufficient sample size, allelic distances of clustered isolate pairs were significantly higher in ARESdb. CgMLST analysis using commercial pipelines may result in different allelic distances but showed concordance using suggested clustering thresholds to determine relatedness among strains.

**IMPORTANCE:**

Microbial genetic relatedness analysis is commonly used to investigate suspected outbreaks among different patients with infections caused by the same species of pathogen and, increasingly, for outbreak surveillance to uncover unsuspected healthcare-associated transmission events among patients, enabling early intervention by infection prevention and control specialists to prevent further spread. Here, we compared three commercial software tools for bacterial relatedness analysis, which perform gene-by-gene comparisons to determine the degree of relatedness for several species of common pathogens. Such software tools potentially allow clinical laboratories to perform rapid and routine analysis for infection control purposes without the need for in-house bioinformatic expertize. This study evaluates the comparability of three of these software tools, while presenting a model for comparative analytic pipeline evaluation.

## INTRODUCTION

Determining whether a healthcare-associated outbreak of a microorganism has occurred conventionally relies upon clinical suspicion and investigation by specialists trained in infection prevention and control. Subsequent investigation of potential outbreaks typically utilizes microbial strain typing to define relatedness among different bacterial or fungal strains isolated in cultures of different patients’ specimens. Previously, strain typing has been performed by slow and imprecise methods such as pulsed-field gel electrophoresis (PFGE) or multilocus sequence typing (MLST), which involves amplification and sequencing of seven housekeeping genes. Many public health and hospital facilities now use whole genome sequencing (WGS), which has higher resolution, to evaluate strain relatedness for outbreak investigation and increasingly for outbreak surveillance (1–3). Analysis of WGS may be performed by different methods. Two common approaches are single nucleotide polymorphism (SNP) analysis, in which every base pair is compared between each isolate and a reference genome, and core genome MLST (cgMLST), in which a set of thousands of predefined “core” genes that are present in all genomes of a given species are analyzed, alleles are assigned at each locus, and subsequent pairwise comparisons of isolates’ allelic profiles are performed (4). CgMLST, compared with SNP analysis, offers a more standardized approach for analyzing more diverse strains that does not rely on defining a closely related reference sequence and has consistent resolution, as the number of targets analyzed is the same for each strain within a species when using the same scheme. However, cgMLST analytic pipelines and schemes may differ in the loci included as well as how alleles are called (4, 5). Determinations of relatedness are based on species-specific genetic distance cutoffs or thresholds, which lack a clear biological basis but have been loosely defined by studies of population genetics or, more commonly, of strain outbreaks with established epidemiologic linkages. Conclusions about strain relatedness could differ depending on the method and thresholds used. In the absence of a reference or “gold-standard” method, we sought to determine the comparability of three commercial cgMLST pipelines for defining relatedness among a subset of commonly isolated bacterial pathogens previously identified by one of the pipelines as potentially related or “clustered” in our comprehensive hospital outbreak surveillance program (1).

## MATERIALS AND METHODS

Isolates were from routine clinical diagnostic cultures collected in years 2020 and 2021 from patients at St. Jude Children’s Research Hospital (SJCRH; Memphis, Tennessee) and were included in this study on the basis of previous identification as being related to at least one other isolate from the same or a different patient using SeqSphere+ (Ridom GmbH, Münster, Germany). For *Acinetobacter baumannii* and *Serratia marcescens,* additional clustering isolates from specimens collected in years 2019 and 2018, respectively, were also included to increase sample sizes for those species. Analyses were performed using publicly available schemes for all species, except *Pseudomonas aeruginosa* and *Serratia marcescens*, which were created *ad hoc* (1). Scheme descriptions, versions, and references are provided in Supplemental Methods (Tables S1 and S2). Species included and clustering thresholds used were from cgMLST.org, a nomenclature server implemented and maintained by Ridom, and are as follows: *Acinetobacter baumannii*: 9*, Escherichia coli*: 10*, Enterococcus faecalis*: 7*, Enterococcus faecium*: 20*, Klebsiella pneumoniae*: 15*, Pseudomonas aeruginosa*: 12*, Staphylococcus aureus:* 24*,* and *Serratia marcescens*: 12. The SJCRH Institutional Review Board designated this study as exempt secondary research; informed consent was not required.

Isolate preparation, DNA extraction, library preparation, Illumina sequencing, and SeqSphere+ analysis were performed as previously described (1). FASTQ files were uploaded and analyzed using the 1928 platform (1928 Diagnostics, Gothenburg, Sweden, accessed December 2023–February 2024) and AREScloud (Ares Genetics GmbH, Opgen, Clarksburg, MD, accessed December 2023–February 2024). In 1928, raw FASTQ data were checked for sufficient quality, including trimming on base quality and primer removal. To detect possible contaminations or mislabeling, built-in species identification is used to confirm species, and multiple alleles in the core genome are detected to identify intra-species contamination. An average coverage of >30 in combination with  ≥95% of good cgMLST targets is considered acceptable sequence quality. The 1928 platform’s cgMLST method uses a custom-developed allele-calling algorithm based on an alignment-free k-mer approach with custom-developed and validated cgMLST schemes created from NCBI RefSeq genomes, wherein those that are present in ≥95% of reference genomes were identified as core genes. Phylogenetic trees are constructed using UPGMA hierarchical clustering, and distance matrices were calculated using all pairwise allelic differences between samples, where missing genes were pairwise ignored. ARESdb analysis consists of *de novo* genome assembly, quality and contamination control, pathogen identification, MLST, and cgMLST analysis with schemes from cgMLST.org. Additional data generated by 1928 and ARESdb including SNP analysis, plasmid replicon typing, virulence marker detection, and detection of antibiotic resistance genes and mutations were not assessed in this study (6, 7).

SeqSphere+, ARESdb, and the 1928 platform were used to generate pairwise matrices of allelic difference of all bacterial isolates included in the data set that could be analyzed. Pairwise allelic differences were compared between pipelines and were evaluated for concordance.

### Statistical analysis

Allelic distance trends across the three categories (same-patient clustered, different-patient clustered, different-patient non-clustered, with “clustered” defined as isolate pairs with SeqSphere+ allelic distances at or below the species-specific thresholds listed above) were evaluated within each pipeline using Jonckheere-Terpstra tests (DescTools package, version 0.99.50), followed by pairwise Mann-Whitney U tests with Bonferroni correction (*n* = 3).

Pipeline comparisons of allelic distances within each category were analyzed initially using Friedman tests to determine whether a significant difference exists among the medians for the three groups. When significant, pairwise Wilcoxon signed-rank tests with Bonferroni correction (*n* = 3) were performed to determine which groups differ and by how much. Effect sizes (r) were calculated as |Z| / √N, where Z was approximated from the *P*-value. Effect size magnitudes were interpreted as small (~0.1), medium (~0.3), or large (~0.5).

Wilcoxon signed-rank tests were used to compare the absolute differences in allelic distances for each pair of pipelines per species for clustered isolates. *P*-values were adjusted using the Benjamini-Hochberg method to control the false discovery rate.

All statistical analyses were conducted in R (version 4.3.0). All statistical tests were two-sided, with a significance level of 0.05. Ties in allelic distances were handled using standard methods, with *P*-values derived from asymptotic approximations: the Jonckheere-Terpstra and Mann-Whitney U tests employed normal approximations with tie-corrected variances; the Wilcoxon signed-rank test assigned average ranks and used a normal approximation with continuity correction; and the Friedman test incorporated a tie correction factor, computing *P*-values from a chi-square approximation.

## RESULTS

This study included a collection of 255 culture isolates of eight species of common bacterial healthcare-associated infection (HAI) -causing pathogens from 97 patients (Table 1). Clustered isolates, or those that were found to be related to at least one other isolate using Ridom SeqSphere+ cgMLST pipeline from our comprehensive WGS-based outbreak surveillance program (1), were included. A total of 6,077 isolate pairs were processed and pairwise distance matrices were generated in SeqSphere+, 1928, and ARESdb. Three *Acinetobacter baumannii*, one *Escherichia coli*, five *Klebsiella pneumoniae*, and one *Pseudomonas aeruginosa* failed analysis by 1928 and/or ARESdb. *Serratia marcescens* was not evaluable by ARESdb because no scheme was available at the time of analysis. These isolates were excluded from the relevant comparisons.

**TABLE 1:** Summary of isolates tested

Species	No. of patients	No. of isolates	Total no. of isolate pairs	No. of isolate pairs by category		
				Same patient	Different patient clustered	Different patient non-clustered
*A. baumannii*	2	6	15	6	0	9
*E. coli*	25	64	2,016	79	2	1,935
*E. faecalis*	9	25	300	27	0	273
*E. faecium*	1	9	36	36	0	0
*K. pneumoniae*	17	45	990	52	65	873
*P. aeruginosa*	14	36	630	41	1	588
*S. aureus*	26	65	2,080	68	9	2,003
*S. marcescens*	3	5	10	2	2	6
Total	97	255	6,077	311	79	5,687

**Fig 1:**
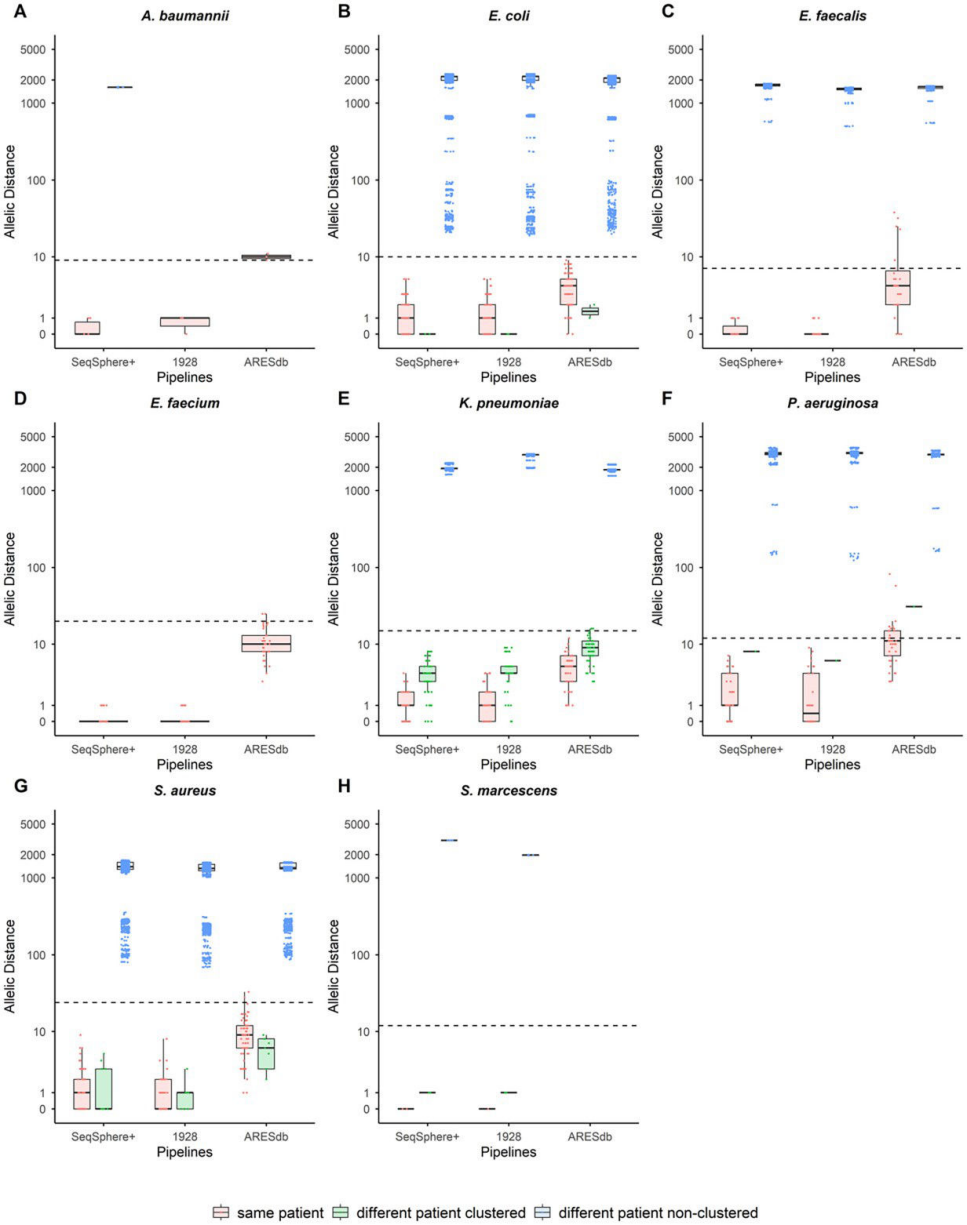
Distribution of allelic distances generated by cgMLST pipelines (SeqSphere+, 1928, and ARESdb) for isolate pairs from eight species, grouped into three categories (‘same-patient’, ‘different-patient clustered’, and ‘different-patient non-clustered’) defined by the SeqSphere+ results. Panels A through H represent individual species: (**A**) *Acinetobacter baumannii*, (**B**) *Escherichia coli*, (**C**) *Enterococcus faecalis*, (**D**) *Enterococcus faecium*, (**E**) *Klebsiella pneumoniae*, (**F**) *Pseudomonas aeruginosa*, (**G**) *Staphylococcus aureus*, and (**H**) *Serratia marcescens*. The broken horizontal line in each panel indicates the suggested SeqSphere+ threshold for differentiating clustered and non-clustered isolates. Red (“same-patient”) or green (“different-patient clustered”) dots above the threshold line, or blue (“different-patient non-clustered”) dots below the threshold line indicate this pipeline’s clustering is inconsistent with SeqSphere+ using the same threshold.

Figure 1 shows the distribution of allelic distances from all three pipelines for isolate pairs of each species. Isolate pairs were categorized into groups in this analysis (“same-patient clustered,” “different-patient clustered,” and “different-patient non-clustered”) by whether they were collected from the same patient or different patients and by whether they were previously found by SeqSphere+ to cluster or not using species-specific thresholds. No same-patient non-clustering isolates were identified by SeqSphere+, nor were any different-patient clustered isolate pairs identified for *Acinetobacter baumannii*, *Enterococcus faecalis*, or *Enterococcus faecium* within the isolate collection (Table 1). Only three same-patient pairs of *A. baumannii* passed analysis in 1928 and ARESdb pipelines (Fig. 1A). For *E. faecium*, all 36 isolate pairs were considered same-patient (Table 1; Fig. 1D).

Clustering agreement between pipelines was evaluated by comparing SeqSphere+ categorizations (clustered versus non-clustered) against 1928 and ARESdb for isolate pairs with available results. All isolate pairs analyzed in the 1928 pipeline produced the same clustered or non-clustered categorization results as SeqSphere+ when applying the same species-specific clustering thresholds (Fig. 1). In contrast, among the 6,055 isolate pairs with both ARESdb and SeqSphere+ results available, ARESdb exhibited 28 inconsistencies with SeqSphere+ in clustering assignments across six species (Fig. 1; Table S3). ARESdb had 99.5% agreement overall with SeqSphere+ and 91.8%, 96.1%, and 100% agreement for isolate pairs in same-patient, different-patient clustered, and different-patient non-clustered categories, respectively (Table S4A). Categorical discrepancies included two same-patient isolate pairs of *A. baumannii*, five same-patient isolate pairs of *E. faecalis*, two same-patient isolate pairs of *E. faecium*, two different-patient clustered isolate pairs of *K. pneumoniae*, 14 same-patient and one different-patient clustered isolate pairs of *P. aeruginosa*, and two same-patient isolate pairs of *S. aureus* (Fig. 1; Table S4B). All different-patient non-clustered isolate pairs had allelic distances greater than the clustering threshold when analyzed by 1928 and ARESdb (Fig. 1; Table S4C).

Trends in allelic distances among relationship categories were investigated within each of the three pipelines. Table 2 shows the pairwise allelic distance comparisons within each relationship category for the 5,747 isolate pairs that were assessed by all three pipelines. Allelic distances for same-patient and different-patient clustered isolate pairs were similar in SeqSphere+ and 1928, but higher in ARESdb. For different patient non-clustered pairs, no such trend in allelic distances emerged as in the other two categories. To evaluate differences in allelic distances across the three categories within each cgMLST pipeline, Jonckheere-Terpstra tests were performed and revealed a significant increasing trend (same patient clustered < different patient clustered < different patient non-clustered) for all three pipelines (*P* < 0.0001 for SeqSphere+, 1928, and ARESdb). Subsequent pairwise Mann-Whitney U tests with Bonferroni correction confirmed that allelic distances for same-patient pairs were significantly lower than those for different-patient clustered pairs (adjusted *P* < 0.0001 for SeqSphere+ and 1928; adjusted *P* = 0.0122 for ARESdb).

**TABLE 2:** Pairwise allelic distance comparisons across three cgMLST pipelines for 5,747 isolate pairs^*a*^

Isolate pair category	No. of isolate pairs	Pipeline	Allelic distance mean (SD)	Allelic distance median (IQR)
Same-patient	300	SeqSphere+	1.18 (1.56)	1 (0, 2)
		1928	1.00 (1.59)	0 (0, 1)
		ARESdb	7.60 (7.17)	6 (3, 10)
Different-patient clustered	67	SeqSphere+	3.61 (2.26)	4 (2, 5)
		1928	3.91 (2.67)	4 (1.5, 5)
		ARESdb	8.34 (4.31)	8 (6, 10)
Different-patient non-clustered	5,380	SeqSphere+	1,731.62 (757.44)	1,849 (1,390.75, 2,210)
		1928	1,824.02 (869.52)	1,896 (1,325, 2,352.25)
		ARESdb	1,674.53 (726.48)	1,776.5 (1,348, 2,108)

^*a*^ SD, standard deviation; IQR, interquartile range.

Differences in allelic distances between pipelines within each category were also compared for the same isolate pairs. For same-patient isolate pairs, a Friedman test indicated significant differences across pipelines (*P* < 0.0001). Subsequent pairwise Wilcoxon signed-rank tests with Bonferroni correction revealed a significant difference between SeqSphere+ and 1928 distances (adjusted *P* = 0.0002) but with a small effect size (r = 0.228). In contrast, ARESdb yielded significantly larger allelic distances than both SeqSphere+ and 1928 (adjusted *P* < 0.0001 for both tests), with large effect sizes (r = 0.855 for ARESdb vs. SeqSphere+ and r = 0.858 for ARESdb vs. 1928).

For different-patient clustered isolate pairs, a Friedman test indicated significant differences in allelic distances across pipelines (*P* < 0.0001). Subsequent pairwise Wilcoxon signed-rank tests with Bonferroni correction indicated SeqSphere+ and 1928 distances have no significant difference (adjusted *P* = 0.0810), while ARESdb produced significantly larger allelic distances than both SeqSphere + and 1928 (adjusted *P* < 0.0001 for both tests), with large effect sizes (r = 0.866 for ARESdb vs. SeqSphere+ and r = 0.870 for ARESdb vs 1928).

For different-patient non-clustered isolate pairs, a Friedman test showed significant differences in allelic distances across pipelines (*P* < 0.0001). Subsequent pairwise Wilcoxon signed-rank tests with Bonferroni correction identified significant differences between SeqSphere+ and 1928, SeqSphere+ and ARESdb, and 1928 and ARESdb (adjusted *P* < 0.0001 for each comparison). However, no pipeline consistently produced larger allelic distances than the other two.

Species-specific differences in allelic distances were evaluated between cgMLST pipelines among the 367 same-patient and different-patient SeqSphere+-clustered isolate pairs that were evaluated in all pipelines (Table 3). For most species, absolute differences in allele distances between 1928 and SeqSphere+ were low, with means ranging from 0 for *A. baumannii* and *E. faecium* to 1.0 for *Pseudomonas aeruginosa* and medians at or near 0. In contrast, absolute differences for ARESdb were larger than SeqSphere+, with means ranging from 2.5 for *Escherichia coli* to 10.8 for *E. faecium* and medians from 2 (*Escherichia coli*) to 9.5 (*E. faecium*). Although not evaluable in ARESdb and thus excluded from the analysis, *S. marcescens* clustered isolates had no difference in allele distances between SeqSphere+ and 1928 pipelines. Wilcoxon signed-rank tests were performed for each of the seven species evaluated in all three pipelines; Benjamini–Hochberg (BH) adjusted *P*-values were less than 0.0001 for all species except *A. baumannii* (*P* = 0.174), which had a small sample size. These results indicate that, for most species, the absolute differences in allelic distances between ARESdb and SeqSphere+ were significantly greater than those between 1928 and SeqSphere+.

**TABLE 3:** Summary of absolute differences in allelic distances between cgMLST pipelines for related (same-patient and different-patient clustered) isolate pairs across seven pathogenic bacterial species analyzed by three pipelines^*a*^

Species	No. of isolate pairs	Difference in allelic distance			
		1928 vs SeqSphere+ mean (SD)	1928 vs SeqSphere+ median (IQR)	ARESdb vs SeqSphere+ mean (SD)	ARESdb vs SeqSphere+ median (IQR)
*Acinetobacter baumannii*	3	0 (0)	0 (0, 0)	9.3 (0.6)	9 (9, 9.5)
*Escherichia coli*	79	0.2 (0.4)	0 (0, 0)	2.5 (1.5)	2 (1, 3)
*Enterococcus faecalis*	27	0.1 (0.4)	0 (0, 0)	7.1 (9.7)	4 (1, 6.5)
*Enterococcus faecium*	36	0 (0)	0 (0, 0)	10.8 (5.5)	9.5 (7, 12.2)
*Klebsiella pneumoniae*	104	0.7 (0.6)	1 (0, 1)	4.2 (1.9)	4 (3, 5)
*Pseudomonas aeruginosa*	41	1.0 (1.2)	1 (0, 1)	10.3 (12)	8 (6, 11)
*Staphylococcus aureus*	77	0.6 (0.9)	0 (0, 1)	7.5 (5.7)	6 (3, 9)

^*a*^ SD, standard deviation; IQR, interquartile range.

## DISCUSSION

While cgMLST has been proposed as a preferred method for standardized strain typing (8, 9), different software pipelines and schemes may not produce identical results. In this study, high concordance between pipelines for assigning clustering versus non-clustering relationships of isolate pairs was found using suggested thresholds, yet further inspection and statistical analysis of the data revealed larger and higher distributions of allelic distances between related isolates in ARESdb compared with those seen with SeqSphere+ and 1928. Use of lower clustering thresholds may lead to lower rates of concordance. The underlying source of variability may be attributable to differences in the methods used for sequence data pre-analytic processing (such as trimming and assembly) and quality control, base calling, the cgMLST schemes used including how genes are assigned and which species, complexes, and strains are included, and/or the allele-calling algorithm used. The specific differences among the pipelines could not be evaluated for their relative impact on the results of this analysis.

Published cgMLST thresholds differ (4, 10) and, when set using epidemiologically confirmed outbreaks, often simply reflect the maximum allelic distance observed among outbreak isolates. Users of these pipelines may choose to define clustering thresholds differently, depending on the goals of the analysis. For example, studying transmission events over long time periods, across many individuals or locations, or through indirect routes may require higher cgMLST cluster thresholds than when used for identifying direct transmission events among few individuals over a short period in a single institution (4). The latter is the primary objective of our hospital-based transmission surveillance efforts, while multi-system, regional, and national public health efforts may take the former approach.

We propose that when WGS-based methods, such as cgMLST, are used routinely at short intervals for hospital outbreak surveillance in the absence of an ongoing outbreak, lower clustering thresholds than those used here with SeqSphere+ or the 1928 platform may be more appropriate. Previous work demonstrated that over multiple years, only 28% of multi-patient clusters identified by routine WGS surveillance had an epidemiologically plausible transmission link identified (1). Multi-patient isolate clusters with epidemiologically plausible links generally showed limited genomic diversity using SeqSphere+ and were typically collected within a short time of each other. These cases included three separate isolate clusters of *S. aureus* with identical cgMLST profiles (0 allelic distance), a fourth cluster comprised of *Klebsiella pneumoniae* isolates with 1–2 allelic differences, and a fifth cluster of *Enterobacter cloacae* isolates with four allelic differences. Epidemiologically plausible links were also seen in a *P. aeruginosa* isolate cluster with seven allelic differences between isolates collected 3 years apart from a patient and from a sink in the adjacent parent room where the patient and their family stayed (11). Unpublished data from ourselves and published data from other authors have demonstrated high reproducibility of cgMLST when using the same pipeline to repeatedly test the same isolate (12), supporting the use of lower thresholds to define relatedness. Future studies may evaluate whether use of lower thresholds in routine genomic surveillance would improve actionability of cluster detections by reducing the number of unsuccessful infection prevention and control investigations. Further gains in outbreak surveillance may be found when microbial genomic surveillance is combined with automated data mining of the electronic health record, which may uncover additional epidemiologic links and reduce the work required of infection preventionists to investigate potential outbreaks among patients with multiple exposures (2, 13).

Serial sampling of isolates from one or more carriers over time has been useful for defining the expected genomic diversity and mutation rates of a given species (5, 14). This information could be used to better define isolate clustering thresholds for species undergoing relatedness analysis. For example, one study found medians of five and two allelic variants per year for *S. aureus* using cgMLST by SeqSphere+ and 1928, respectively (5), while the cgMLST clustering threshold used here was 24, which may be more appropriate for extensive and prolonged outbreaks. However, some strains exhibit elevated mutation frequencies or hypermutator phenotypes, such as those with defects in DNA repair or when grown under certain conditions (15, 16), so some caution should be applied when interpreting clustering results with low thresholds and repeated sampling from colonized or infected patients may be required. Further characterization of analytic tools and thresholds with strains containing hypermutator phenotypes may be beneficial.

The present study had certain limitations. Included isolates represented a subset of those collected over the noted timeframe, enriched for isolates that demonstrated relatedness to one another using recommended clustering thresholds. Inclusion of additional non-clustering isolates would allow further characterization of specificity of different pipelines for outbreak detection. We were unable to compare relatedness determinations among pipelines for less common HAI pathogens that have produced outbreaks in our setting and others (17–19), as both 1928 and ARESdb lack pre-defined schemes to enable analysis of these species or to create *ad hoc* schemes for this purpose. This study encompasses three commercial analytic pipelines, available at the time of study, as a representation of commercial products in this area. ARESdb is not currently operational at the time of this writing, and new products may well have entered the market. This study is not a comprehensive assessment of products on the market, which vary over time, but does provide a useful comparison of analytic products made available for outbreak investigation or surveillance and a model for comparison of such pipelines going forward.

WGS-based microbial genetic relatedness analysis is rapidly expanding and becoming more routine in healthcare and public health institutions, as a valuable component of infection prevention and control investigations and surveillance (20, 21). The availability of commercial software pipelines that perform cgMLST alleviates the need for in-house bioinformatics expertize, a significant burden for most clinical laboratories, and a major limitation to performing rapid and routine analysis. Evaluation of pipelines will be an ongoing challenge, just as it is for pathogen detection platforms. Future work should address further validating and standardizing cgMLST analysis and thresholds for relatedness for common bacterial pathogens, while continuing to evaluate the accuracy and comparability of analytic products.

## Data Availability

Sequences from isolates in this study are available in the National Center for Biotechnology (NCBI) Sequence Read Archive (SRA) under accession no. PRJNA1287502.

## SUPPLEMENTAL MATERIAL

Supplemental tables jcm.00646-25-s0001.docx Tables S1 to S4.

ASM does not own the copyrights to Supplemental Material that may be linked to, or accessed through, an article. The authors have granted ASM a non-exclusive, world-wide license to publish the Supplemental Material files. Please contact the corresponding author directly for reuse.

## References

[1] Hakim H, Glasgow HL, Brazelton JN, Gilliam CH, Richards L, Hayden RT. 2024. A prospective bacterial whole-genome-sequencing-based surveillance programme for comprehensive early detection of healthcare-associated infection transmission in paediatric oncology patients. J Hosp Infect 143:53–63. doi:10.1016/j.jhin.2023.10.015 37939882

[2] Sundermann AJ, Chen J, Kumar P, Ayres AM, Cho ST, Ezeonwuka C, Griffith MP, Miller JK, Mustapha MM, Pasculle AW, Saul MI, Shutt KA, Srinivasa V, Waggle K, Snyder DJ, Cooper VS, Van Tyne D, Snyder GM, Marsh JW, Dubrawski A, Roberts MS, Harrison LH. 2022. Whole-genome sequencing surveillance and machine learning of the electronic health record for enhanced healthcare outbreak detection. Clin Infect Dis 75:476–482. doi:10.1093/cid/ciab946 34791136 PMC9427134

[3] Mellmann A, Bletz S, Böking T, Kipp F, Becker K, Schultes A, Prior K, Harmsen D. 2016. Real-time genome sequencing of resistant bacteria provides precision infection control in an institutional setting. J Clin Microbiol 54:2874–2881. doi:10.1128/JCM.00790-16 27558178 PMC5121374

[4] Schürch AC, Arredondo-Alonso S, Willems RJL, Goering RV. 2018. Whole genome sequencing options for bacterial strain typing and epidemiologic analysis based on single nucleotide polymorphism versus gene-by-gene-based approaches. Clin Microbiol Infect 24:350–354. doi:10.1016/j.cmi.2017.12.016 29309930

[5] Lagos AC, Sundqvist M, Dyrkell F, Stegger M, Söderquist B, Mölling P. 2022. Evaluation of within-host evolution of methicillin-resistant Staphylococcus aureus (MRSA) by comparing cgMLST and SNP analysis approaches. Sci Rep 12:10541. doi:10.1038/s41598-022-14640-w 35732699 PMC9214674

[6] Ferreira I, Beisken S, Lueftinger L, Weinmaier T, Klein M, Bacher J, Patel R, von Haeseler A, Posch AE. 2020. Species identification and antibiotic resistance prediction by analysis of whole-genome sequence data by use of ARESdb: an analysis of isolates from the unyvero lower respiratory tract infection trial. J Clin Microbiol 58:e00273-20. doi:10.1128/JCM.00273-20 32295890 PMC7315026

[7] Ferreira I, Lepuschitz S, Beisken S, Fiume G, Mrazek K, Frank BJH, Huber S, Knoll MA, von Haeseler A, Materna A, Hofstaetter JG, Posch AE, Weinberger J. 2021. Culture-free detection of antibiotic resistance markers from native patient samples by hybridization capture sequencing. Microorganisms 9:1672. doi:10.3390/microorganisms9081672 34442751 PMC8398375

[8] Nadon C, Van Walle I, Gerner-Smidt P, Campos J, Chinen I, Concepcion-Acevedo J, Gilpin B, Smith AM, Man Kam K, Perez E, Trees E, Kubota K, Takkinen J, Nielsen EM, Carleton H, FWD-NEXT Expert Panel. 2017. PulseNet international: vision for the implementation of whole genome sequencing (WGS) for global food-borne disease surveillance. Euro Surveill 22:30544. doi:10.2807/1560-7917.ES.2017.22.23.30544 28662764 PMC5479977

[9] Leopold SR, Goering RV, Witten A, Harmsen D, Mellmann A. 2014. Bacterial whole-genome sequencing revisited: portable, scalable, and standardized analysis for typing and detection of virulence and antibiotic resistance genes. J Clin Microbiol 52:2365–2370. doi:10.1128/JCM.00262-14 24759713 PMC4097726

[10] Siddall RL, Starkey JC, Patel R. 2025. Automated whole genome sequencing platform for bacterial strain typing in clinical microbiology laboratories. J Clin Microbiol 63:e0017825. doi:10.1128/jcm.00178-25 40261051 PMC12077132

[11] Richards L, Gilliam C, Brazelton J, Glasgow HL, Hayden RT, Hakim H. 2025. A persistent sink reservoir as a potential source of Pseudomonas aeruginosa infections in pediatric oncology patients. Antimicrob Steward Healthc Epidemiol 5:e82. doi:10.1017/ash.2025.54 40160224 PMC11951235

[12] Mellmann A, Andersen PS, Bletz S, Friedrich AW, Kohl TA, Lilje B, Niemann S, Prior K, Rossen JW, Harmsen D. 2017. High interlaboratory reproducibility and accuracy of next-generation-sequencing-based bacterial genotyping in a ring trial. J Clin Microbiol 55:908–913. doi:10.1128/JCM.02242-16 28053217 PMC5328459

[13] Sundermann AJ, Miller JK, Marsh JW, Saul MI, Shutt KA, Pacey M, Mustapha MM, Ayres A, Pasculle AW, Chen J, Snyder GM, Dubrawski AW, Harrison LH. 2019. Automated data mining of the electronic health record for investigation of healthcare-associated outbreaks. Infect Control Hosp Epidemiol 40:314–319. doi:10.1017/ice.2018.343 30773168 PMC8189294

[14] Mustapha MM, Srinivasa VR, Griffith MP, Cho ST, Evans DR, Waggle K, Ezeonwuka C, Snyder DJ, Marsh JW, Harrison LH, Cooper VS, Van Tyne D. 2022. Genomic diversity of hospital-acquired infections revealed through prospective whole-genome sequencing-based surveillance. mSystems 7:e0138421. doi:10.1128/msystems.01384-21 35695507 PMC9238379

[15] Köser CU, Holden MTG, Ellington MJ, Cartwright EJP, Brown NM, Ogilvy-Stuart AL, Hsu LY, Chewapreecha C, Croucher NJ, Harris SR, Sanders M, Enright MC, Dougan G, Bentley SD, Parkhill J, Fraser LJ, Betley JR, Schulz-Trieglaff OB, Smith GP, Peacock SJ. 2012. Rapid whole-genome sequencing for investigation of a neonatal MRSA outbreak. N Engl J Med 366:2267–2275. doi:10.1056/NEJMoa1109910 22693998 PMC3715836

[16] Cabot G, Zamorano L, Moyà B, Juan C, Navas A, Blázquez J, Oliver A. 2016. Evolution of Pseudomonas aeruginosa antimicrobial resistance and fitness under low and high mutation rates. Antimicrob Agents Chemother 60:1767–1778. doi:10.1128/AAC.02676-15 26729493 PMC4775977

[17] Tönnies H, Heep A, Herrmann J, Lange M, Mellmann A, Hamprecht A. 2024. Investigating environmental transmission to resolve a Bacillus cereus group outbreak in a neonatal intensive care unit using core genome multilocus sequence typing. Antimicrob Resist Infect Control 13:1. doi:10.1186/s13756-023-01359-0 38184647 PMC10771705

[18] Gilliam CH, Brazelton de Cardenas J, Carias D, Maron Alfaro G, Hayden RT, Hakim H. 2023. Lactobacillus bloodstream infections genetically related to probiotic use in pediatric hematopoietic cell transplant patients. Infect Control Hosp Epidemiol 44:484–487. doi:10.1017/ice.2021.515 35225182

[19] Roach DJ, Burton JN, Lee C, Stackhouse B, Butler-Wu SM, Cookson BT, Shendure J, Salipante SJ. 2015. A year of infection in the intensive care unit: prospective whole genome sequencing of bacterial clinical isolates reveals cryptic transmissions and novel microbiota. PLoS Genet 11:e1005413. doi:10.1371/journal.pgen.1005413 26230489 PMC4521703

[20] Sherry NL, Gorrie CL, Kwong JC, Higgs C, Stuart RL, Marshall C, Ballard SA, Sait M, Korman TM, Slavin MA, Lee RS, Graham M, Leroi M, Worth LJ, Chan HT, Seemann T, Grayson ML, Howden BP, Controlling Superbugs Study G. 2022. Multi-site implementation of whole genome sequencing for hospital infection control: a prospective genomic epidemiological analysis. Lancet Reg Health West Pac 23:100446. doi:10.1016/j.lanwpc.2022.100446 35465046 PMC9019234

[21] Peacock SJ, Parkhill J, Brown NM. 2018. Changing the paradigm for hospital outbreak detection by leading with genomic surveillance of nosocomial pathogens. Microbiology 164:1213–1219. doi:10.1099/mic.0.000700 30052172 PMC7611365

