# Supplementary material for: Comparison of core genome multi-locus sequencing typing pipelines for hospital outbreak detection of common bacterial pathogens
Source: J Clin Microbiol. 2025 Aug 27;63(10):e00646-25. doi: 10.1128/jcm.00646-25 (PMC12506026; doi:10.1128/jcm.00646-25)
Supplement: Supplemental tables — Tables S1 to S4. [file jcm.00646-25-s0001.docx]

**Supplemental Methods**

**Supplemental Table 1. SeqSphere+ schemes used in this study**

| Organism | Scheme | Public (cgMLST.org)/ ad hoc | References/Description | Version |
| --- | --- | --- | --- | --- |
| *Acinetobacter baumannii* | *A. baumannii* cgMLST | Public | PubMed: 28594944 | 1 |
| *Escherichia coli* | *E. coli* cgMLST | Public | This Escherichia coli core genome MLST scheme uses exactly the same loci and reference gene sequences as the EnteroBase (http://enterobase.warwick.ac.uk) Escherichia/Shigella cgMLST v1 scheme. However, the SeqSphere+ allele calling procedure is slightly different and our allele numbering is independent from and not compatible with the EnteroBase allele nomenclature. | 1 |
| *Enterococcus faecalis* | *E. faecalis* cgMLST | Public | PubMed: 30651394 | 1 |
| *Enterococcus faecium* | *E. faecium* cgMLST | Public | PubMed: 26400782 | 1.1 |
| *Klebsiella pneumoniae* | *K. pneumoniae* *sensu lato* cgMLST | Public | Not available | 1 |
| *Pseudomonas aeruginosa* | *P. aeruginosa* cgMLST 4073 targets PAO1 | Ad hoc | PubMed: 37939882 | 1 |
| *Serratia marcescens* | *S. marcescens* cgMLST 3219 targets Db11 | Ad hoc | PubMed: 37939882 | 1 |
| *Staphylococcus aureus* | *S. aureus* cgMLST | Public | PubMed: 24759713 | 1.3 |

**Supplemental Table 2. 1928 schemes used in this study**

| Organism | cgMLST base reference | Core genes |
| --- | --- | --- |
| *Acinetobacter baumannii* | XH858 | 2308 |
| *Enterococcus faecalis* | VE18395 | 1770 |
| *Enterococcus faecium* | Aus0085 | 1315 |
| *Escherichia coli* | JEONG-1266 | 2510 |
| *Klebsiella pneumoniae* | KP69 | 3459 |
| *Pseudomonas aeruginosa* | NCGM257 | 3937 |
| *Serratia marcescens* | ATCC 13880 | 2124 |
| *Staphylococcus aureus* | Newman | 1704 |

**Supplemental Results**

**Supplemental Table 3.** Allelic distances and clustering results for the 28 inconsistent isolate pairs

| **Species** | **Species clustering threshold** | **Isolate pair category (SeqSphere+)** | **Allelic distance**  **1928** | **Allelic distance**  **ARESdb** | **Allelic distance**  **SeqSphere+** | **Relationship assignment**  **1928** | **Relationship assignment**  **ARESdb** | **Relationship assignment**  **SeqSphere+** |
| --- | --- | --- | --- | --- | --- | --- | --- | --- |
| *A. baumannii* | 9 | same-patient clustered | 1 | 10 | 1 | related | unrelated | related |
| *A. baumannii* | 9 | same-patient clustered | 1 | 11 | 1 | related | unrelated | related |
| *E. faecalis* | 7 | same-patient clustered | 0 | 25 | 0 | related | unrelated | related |
| *E. faecalis* | 7 | same-patient clustered | 0 | 32 | 1 | related | unrelated | related |
| *E. faecalis* | 7 | same-patient clustered | 0 | 38 | 1 | related | unrelated | related |
| *E. faecalis* | 7 | same-patient clustered | 0 | 9 | 0 | related | unrelated | related |
| *E. faecalis* | 7 | same-patient clustered | 0 | 23 | 1 | related | unrelated | related |
| *E. faecium* | 20 | same-patient clustered | 0 | 25 | 0 | related | unrelated | related |
| *E. faecium* | 20 | same-patient clustered | 0 | 25 | 0 | related | unrelated | related |
| *K. pneumoniae* | 15 | different-patient clustered | NA | 16 | 8 | NA | unrelated | related |
| *K. pneumoniae* | 15 | different-patient clustered | NA | 16 | 7 | NA | unrelated | related |
| *P. aeruginosa* | 12 | same-patient clustered | 0 | 17 | 4 | related | unrelated | related |
| *P. aeruginosa* | 12 | same-patient clustered | 0 | 13 | 0 | related | unrelated | related |
| *P. aeruginosa* | 12 | same-patient clustered | 0 | 20 | 2 | related | unrelated | related |
| *P. aeruginosa* | 12 | same-patient clustered | 9 | 13 | 6 | related | unrelated | related |
| *P. aeruginosa* | 12 | same-patient clustered | 0 | 82 | 2 | related | unrelated | related |
| *P. aeruginosa* | 12 | same-patient clustered | NA | 58 | 2 | NA | unrelated | related |
| *P. aeruginosa* | 12 | same-patient clustered | 4 | 15 | 5 | related | unrelated | related |
| *P. aeruginosa* | 12 | same-patient clustered | 5 | 16 | 6 | related | unrelated | related |
| *P. aeruginosa* | 12 | same-patient clustered | 4 | 15 | 5 | related | unrelated | related |
| *P. aeruginosa* | 12 | same-patient clustered | 5 | 15 | 6 | related | unrelated | related |
| *P. aeruginosa* | 12 | same-patient clustered | 5 | 17 | 7 | related | unrelated | related |
| *P. aeruginosa* | 12 | same-patient clustered | 0 | 15 | 1 | related | unrelated | related |
| *P. aeruginosa* | 12 | same-patient clustered | 0 | 16 | 1 | related | unrelated | related |
| *P. aeruginosa* | 12 | same-patient clustered | 0 | 17 | 1 | related | unrelated | related |
| *P. aeruginosa* | 12 | different-patient clustered | 6 | 31 | 8 | related | unrelated | related |
| *S. aureus* | 24 | same-patient clustered | 0 | 28 | 0 | related | unrelated | related |
| *S. aureus* | 24 | same-patient clustered | 0 | 33 | 1 | related | unrelated | related |

**Supplemental Table 4A.** ARESdb and SeqSphere+ agreement by clustering category for 6055 isolate pairs

| **Category** | **Isolate pairs with clustering assignment agreement (n)** | **Isolate pairs with clustering assignment inconsistency (n)** | **Total (n)** | **Percentage agreement** |
| --- | --- | --- | --- | --- |
| same-patient clustered | 281 | 25 | 306 | 91.8% |
| different-patient clustered | 74 | 3 | 77 | 96.1% |
| different-patient non-clustered | 5672 | 0 | 5672 | 100% |

**Supplemental Table 4B.** ARESdb and SeqSphere+ agreement by species for 6055 isolate pairs

| **Species** | **Isolate pairs with clustering assignment agreement (n)** | **Isolate pairs with clustering assignment inconsistency (n)** | **Total (n)** | **Percentage agreement** |
| --- | --- | --- | --- | --- |
| *A. baumannii* | 1 | 2 | 3 | 33.3% |
| *E. coli* | 2016 | 0 | 2016 | 100% |
| *E. faecalis* | 295 | 5 | 300 | 98.3% |
| *E. faecium* | 34 | 2 | 36 | 94.4% |
| *K. pneumoniae* | 988 | 2 | 990 | 99.8% |
| *P. aeruginosa* | 615 | 15 | 630 | 97.6% |
| *S. aureus* | 2078 | 2 | 2080 | 99.9% |

**Supplemental Table 4C.** ARESdb and SeqSphere+ agreement by species and category for 6055 isolate pairs

| **Species** | **Category** | **Isolate pairs with clustering assignment agreement (n)** | **Isolate pairs with clustering assignment inconsistency (n)** | **Total (n)** | **Percentage agreement** |
| --- | --- | --- | --- | --- | --- |
| *A. baumannii* | same-patient clustered | 1 | 2 | 3 | 33.3% |
| *E. coli* | same-patient clustered | 79 | 0 | 79 | 100% |
|  | different-patient clustered | 2 | 0 | 2 | 100% |
|  | different-patient non-clustered | 1935 | 0 | 1935 | 100% |
| *E. faecalis* | same-patient clustered | 22 | 5 | 27 | 81.5% |
|  | different-patient non-clustered | 273 | 0 | 273 | 100% |
| *E. faecium* | same-patient clustered | 34 | 2 | 36 | 94.4% |
| *K. pneumoniae* | same-patient clustered | 52 | 0 | 52 | 100% |
|  | different-patient clustered | 63 | 2 | 65 | 96.9% |
|  | different-patient non-clustered | 873 | 0 | 873 | 100% |
| *P. aeruginosa* | same-patient clustered | 27 | 14 | 41 | 65.9% |
|  | different-patient clustered | 0 | 1 | 1 | 0% |
|  | different-patient non-clustered | 588 | 0 | 588 | 100% |
| *S. aureus* | same-patient clustered | 66 | 2 | 68 | 97.1% |
|  | different-patient clustered | 9 | 0 | 9 | 100% |
|  | different-patient non-clustered | 2003 | 0 | 2003 | 100% |
